# The role of NUDT21 in microRNA‐binging sites of EZH2 gene increases the of risk preeclampsia

**DOI:** 10.1111/jcmm.14179

**Published:** 2019-03-18

**Authors:** Xiao Lang, Wenxia Zhao, Ding Huang, Wei Liu, Hong Shen, Liang Xu, Shan Xu, Yongfang Huang, Weiwei Cheng

**Affiliations:** ^1^ Department of Obstetrics and Gynecology, School of Medicine International Peace Maternity and Child Health Hospital, Shanghai Jiao Tong University Shanghai China; ^2^ Department of Obstetrics and Gynecology Jiangwan Hospital of Shanghai Hongkou District Shanghai China

**Keywords:** alternative polyadenylation, EZH2, microRNA, NUDT21, preeclampsia

## Abstract

**Objectives:**

Preeclampsia (PE) is a major cause of mortality and morbidity among pregnant mothers and their fetuses worldwide. Recent studies have shown that several microRNAs (miRNAs) play crucial role in pathogenesis of PE patients; however, the mechanisms responsible for differences in miRNA function in PE largely remain to be determined.

**Materials and Methods:**

We studied that NUDT21 expression was markedly increased, whereas EZH2 was decreased in placental samples from patients with PE. We identified NUDT21 as an interaction partner of enhancer of zeste homologue 2 (EZH2). NUDT21 co‐localized with EZH2 in the human trophoblast cell line, HTR‐8/SVneo and NUDT21 was shown to bind to EZH2 in RNA immunoprecipitation assays. NUDT21 has previously been reported to be involved in alternative polyadenylation; thus, the interaction between NUDT21 and EZH2 may play an important role in the crosstalk between alternative polyadenylation (APA) and miRNA‐mediated gene silencing in PE.

**Results:**

In the human trophoblast cell line HTR‐8/SVneo, loss‐of‐function assays indicated that knockdown of NUDT21 suppressed cell proliferation, migration and tube formation. Furthermore, functional studies showed that NUDT21 elongated the 3'‐UTR of mRNAs thereby exposing more miRNA binding sites (including miR138 and miR363), which enhanced the efficiency of miRNA‐mediated gene silencing and promoted EZH2 binding.

**Conclusions:**

This is the first report about the relationship of NUDT21 and EZH2. The data indicate that the aberrant expression of NUDT21 contributes to PE by targeting 3'‐UTR of EZH2 mRNA. These findings may provide novel targets for future investigations into therapeutic strategies for PE.

## 1 INTRODUCTION

Preeclampsia (PE) is a condition that affects 2%‐8% of pregnant women worldwide during the second half of their pregnancy and is characterized by hypertension, proteinuria and edema and in severe cases, organ damage. The placenta of a PE patient develops abnormally due to poor trophoblastic invasion of the uterine spiral arteries, leading to oxidative stress, hypoxia, secretion of inflammatory mediators, neutrophil activation and endothelial dysfunction.^1^ However, much of the pathophysiology of PE remains to be determined.

MicroRNAs (miRNAs) are small, single‐strand RNAs that play a crucial role in the posttranscriptional regulation of gene expression. MiRNAs repress the expression of several genes simultaneously by binding to and either inhibiting or cleaving their target mRNAs, with as much as 30% of the human genome thought to be regulated by miRNAs^2^ and around 60% of human protein coding genes being regulated by miRNAs.^3^ Previous studies have identified a number of miRNAs that are specifically expressed in placenta tissue.^4^, ^5^, ^6^ Furthermore, recent studies have revealed that distinct subsets of miRNAs are expressed differentially in the human placentas of patients with PE compared with normal pregnant controls.^7^, ^8^, ^9^ However, the mechanisms responsible for mediating the functions of these miRNAs in PE remain to be elucidated.

Alternative polyadenylation is one mechanism of gene expression regulation that gives rise to a variety of transcripts from a single gene. The presence in a gene of multiple polyadenylation sites (poly(A) sites), leads to changes in 3ʹ‐untranslated region (UTR) lengths and thereby variable mRNA and protein products. The consequence of these variable transcript lengths is that RNA‐binding proteins and/or miRNAs may bind differentially to the longer or shorter isoforms.^10^ The 3ʹ‐UTR length is therefore crucial for efficient miRNA‐mediated gene silencing. This has been widely documented in the case of oncogenes.^11^, ^12^ For example, in hepatocellular carcinoma (HCC), it was previously shown that shortening of the 3'‐UTRs of various oncogenes effectively removed miRNA binding sites enabling these genes to evade miRNA regulation, become overexpressed, and induce tumorigenesis and metastasis.^13^

NUDT21 (also known as CPSF5 and CFIM25) is a highly conserved hydrolase that forms one subunit of the cleavage factor Im complex (CFIM) required for 3' RNA cleavage and polyadenylation processing, which are early steps in the assembly of eukaryotic pre‐mRNA.^14^, ^15^ It has previously been shown that knockdown of NUDT21 leads to changes in alternative poly(A) site utilization promoting the selection of the proximal ploy(A) site and thereby shortening the 3'‐UTRs of several somatic mRNAs.^16^, ^17^

Enhancer of zeste homologue 2 (EZH2) functions as a histone–lysine N‐methyltransferase and is the catalytic subunit of the transcriptional repressor polycomb repressive complex 2 (PRC2). Following histone methylation (primarily of histone H3 on lysine 27), EZH2 recruits and binds polycomb repressive complex 1 (PRC1), which facilitates transcriptional repression and maintains silencing of the target genes.^18^, ^19^, ^20^, ^21^, ^22^ The PRC protein complex is highly conserved among organisms and binds to and represses transcription of many developmentally important regulators involved in stem cell differentiation and early embryonic development.^23^, ^24^

In this study, we investigated the molecular basis for the marked increase in NUDT21 expression in placental samples from patients with PE. Our results indicated that NUDT21 promotes the generation of longer mRNA transcripts, thereby exposing more miRNA binding sites and enhancing miRNA‐mediated gene silencing of EZH2, leading to PE development and progression. Our findings therefore provide novel insight into the pathophysiology of PE.

## 2 MATERIALS AND METHODS

### 2.1 Tissue samples and cell culture

Twenty paired placental tissues from PE women, who were diagnosed with PE and underwent deliveries at International Peace Maternity and Child Health Hospital between 2016 and 2018, and women with normal pregnancies were collected. All tissue samples were immediately frozen in liquid nitrogen and stored at −80°C prior to RNA extraction.

Two cell lines were used in this study: the human trophoblast cell line HTR‐8/SVneo, and the HUVEC‐C cell line, which was obtained from the Type Culture Collection of the Chinese Academy of Sciences (Shanghai, China). HTR‐8/SVneo cells were cultured in RPMI 1640 and HUVEC‐C cells were cultured in ECM (KeyGEN, Nanjing, China), to which 10% fetal bovine serum (GIBCO, BRL, Invitrogen, Carlsbad, CA, USA),100 U/mL penicillin and 100 mg/mL streptomycin (Invitrogen) were added. Cells were incubated in a humidified atmosphere of 5% CO_2_ at 37°C and were passaged every 3 days.

### 2.2 Plasmid construction

The full‐length 3ʹ‐UTR of EZH2 was inserted into the pGL3‐Control luciferase vector (Promega, Madison, WI, USA) following the manufacturer's instructions. The reporter expression vectors were used as an internal control for the dual‐luciferase assay. SiNUDT21 and empty vector negative controls were obtained from Santa Cruz Biotechnology, Santa Cruz, CA, USA.

### 2.3 Immunostaining and immunohistochemistry (IHC)

For the immunostaining of cells, cells were first seeded onto gelatin‐pre‐treated slides and were then fixed with 4% PFA. After blocking with 10% horse serum in PBS plus 3%, the slides were incubated with the appropriate anti‐NUDT21 and anti‐EZH2 primary antibodies and Alexa Fluor 488‐ or 594‐conjugated secondary antibodies (Jackson ImmunoResearch, West Grove, USA). Cell nuclei were detected with DAPI (Invitrogen) then imaged under a confocal microscope (Tcs‐sp5‐II; Leica, Wetzlar, Germany).

For the immunohistochemical detection of proteins in tumour tissue, placental tissue samples were fixed with 4% PFA, dehydrated and then embedded in paraffin. The paraffin‐embedded tissues were then sectioned using a microtome (RM2245; Leica), and the slide‐mounted sections were deparaffinized and rehydrated. Antigens were retrieved by microwaving in sodium citrate buffer (10 mmol/L sodium citrate acid, 0.05% Tween 20, pH 6.0) for 2 minutes. After blocking with 3% H_2_O_2_, followed by normal human serum for 1 hour, the sections were subjected to immunohistochemical staining using the appropriate anti‐NUDT21 and anti‐EZH2 primary antibodies and HRP‐conjugated secondary antibodies. The signals were then detected with DAB substrate (ZSGB‐BIO, Beijing, China), and the sections were counterstained with hematoxylin.

### 2.4 RNA preparation and qRT‐PCR

Total RNA was extracted from cells using TRIzol reagent (Life Technologies) and quantified using a NanoDrop spectrophotometer (2000c; Thermo Fisher Scientific). Then, 1 μg of RNA was reverse transcribed in a reaction volume of 20 μL using the PrimeScript RT reagent kit (TaKaRa, Dalian, China) and SYBR Premix Ex Taq (TaKaRa). qRT‐PCR assays were performed using an ABI 7500 (Invitrogen). The expression of NUDT21 and EZH2 was evaluated using the following primer pairs: NUDT21: F 5ʹ‐TCGGCAACAAGTACATCCAGC‐3ʹ and R 5ʹ‐CGCTGAAATCT GGCTGCAACA‐3ʹ; EZH2: F 5ʹ‐CTTGGTCTCCCCTACAGCAGA‐3ʹ and R 5ʹ‐ACCCACATTCTCTATCCCCGT‐3ʹ, proximal EZH2: F 5ʹ‐AATCCCTTGAC ATCTGCT‐3ʹ and R 5ʹ‐TTTCTAAATTGCCCACAG‐3ʹ; distal EZH2: F 5ʹ‐GCAAAGTACTGTAAGAAT‐3ʹ and R 5ʹ‐TCACTGGTACAAAACAC T‐3ʹ and normalized to GAPDH as an endogenous control. The qPCR results were analysed and expressed relative to the threshold cycle (CT) values and presented as fold changes.

### 2.5 Western blot analysis

Transfected cells were lysed in lysis buffer^25^ containing a protease inhibitor cocktail (Sigma) and the proteins (25 µg) were separated by 10% SDS–PAGE, then electro‐transferred onto PVDF membrane (Merck Millipore, Darmstadt, Germany). After blocking in 5% skim milk at room temperature for 1 hour, membranes were incubated with primary antibodies anti‐NUDT21 (sc‐81109; Santa Cruz) or anti‐EZH2 (#5246; Cell signaling) at 4°C overnight, followed by the appropriate secondary antibodies conjugated with HRP. Immunoreactive protein signals were detected using Pierce ECL substrate (Life Technologies) and a ChemiDoc MP System (BioRad). Densitometric analysis of the bands was performed using ImageJ software (National Institute of Health, USA).

### 2.6 Cell viability assay and colony formation test

To assess the number of live cells in cell culture, a 3‐[4, 5dimethylthiazol‐2‐yl]‐2, 5‐dimethyltetrazolium‐bromide (MTT, Invitrogen, cat no. M6494) assay was performed. Briefly, cells were seeded in 200 µL of media in 96‐well plates (5 × 10^3^ cells/well) and incubated at 37°C for 24 hours. Then, 20 μL of sterile MTT (5 mg/mL; Sigma) was added and incubation was continued for 4 hours at 37°C. To stop the reaction, the culture media was removed and 150 μL of dimethyl sulphoxide (DMSO) was added. The absorbance was then measured on an ELISA reader (BioTek, Vermont, VT, USA) at wavelengths of 490 nm. All reactions were performed in triplicate.

Cells were plated at a density of 5 × 10^3^ cells/well in a 6‐well plate in DMEM culture medium containing 10% FBS. The medium was replaced every 4 or 5 days. After 21 days, colonized were fixed with 4% paraformaldehyde in PBS containing 4% sucrose for 20 minutes, and then stained with 0.005% crystal violet for 30 minutes at room temperature. Then, they were washed three times with PBS for 5 minutes. The relative percentage of positive cells was examined from three to five subjects in three wells.

### 2.7 Cell migration and invasion assays

Cell migration and invasion assays were performed as previously reported.^26^ Both assays followed a similar protocol except that the transwell insert (Costar, Cambridge, MA, USA) was pre‐coated with Matrigel (BD Biosciences, Beit‐Ha’ Emek, Israel) for the cell invasion assay but not for the cell migration assay. Briefly, 3‐5×10^4^ HTR‐8/SVneo cells were cultivated in media without FBS in the upper chamber of the transwell insert, whereas media containing FBS was added to the lower chamber. After incubation at 37°C for 24, 36, 48 and 60 hours, cells in the upper chamber were removed using a cotton swab whereas cells on the lower surface of the membrane were fixed with pre‐chilled methanol and then stained with 0.5% crystal violet solution. Stained cells were visualized under a microscope and five randomly selected fields were counted per well to determine the number of invading or migrating cells relative to the corresponding control.

### 2.8 In vitro angiogenesis assay

The primary human umbilical vein endothelial cells were collected from umbilical cords by collagenase perfusion of the umbilical vein, and the isolated cells were then seeded into 6‐well plates at a concentration of 2.4 × 10^5^ cells/well using EBM‐2 me‐dium (CC‐3156, Lonza, USA) and incubated at 37℃ in a 5% CO_2_ and 95% atmosphere overnight. The In vitro Angiogenesis Assay Kit (Cat no. 3470‐096‐K, Trevigen, Gaithersburg, MD, USA) was employed. The total capillary tube length was determined for each well using the Angioquant image analysis software.^27^

### 2.9 RNA immunoprecipitation assays

To investigate the interaction between NUDT21 and EZH2, the Magna RIPTM RNA‐Binding Protein Immunoprecipitation Kit (Merck Millipore) was used along with NUDT21 antibody (or an IgG control) to pull down EZH2. This procedure was carried out according to the manufacturer's instructions. Protein A/G beads were used to recover the antibody. Then, qRT‐PCR was performed using standard protocols to detect EZH2 mRNA in the precipitates.

### 2.10 Dual‐luciferase assay

A EZH2 luciferase reporter system was constructed by co‐transfecting HTR‐8/SVneo cells with the wild‐type or mutant EZH2 3ʹ‐UTR‐containing vector and pRenilla‐TK, together with miR‐138‐5p or miR‐363‐5p mimics or the corresponding control miRNA. After 48 hours, luciferase activity was assessed using the Dual‐Luciferase Reporter Assay Kit (Promega) and a Varioskan™ Flash Microplate Reader.

### 2.11 Statistical analysis

Differences between groups were analysed statistically using the Student's *t* test (SPSS Statistics 17.0, Chicago, IL, USA). All data are expressed as the mean ± standard deviation (SD) based on at least three independent experiments. *P *< 0.05 or 0.01 indicated statistically significant differences.

## 3 RESULTS

### 3.1 NUDT21 expression levels are increased in PE placental tissue

**Figure 1:**
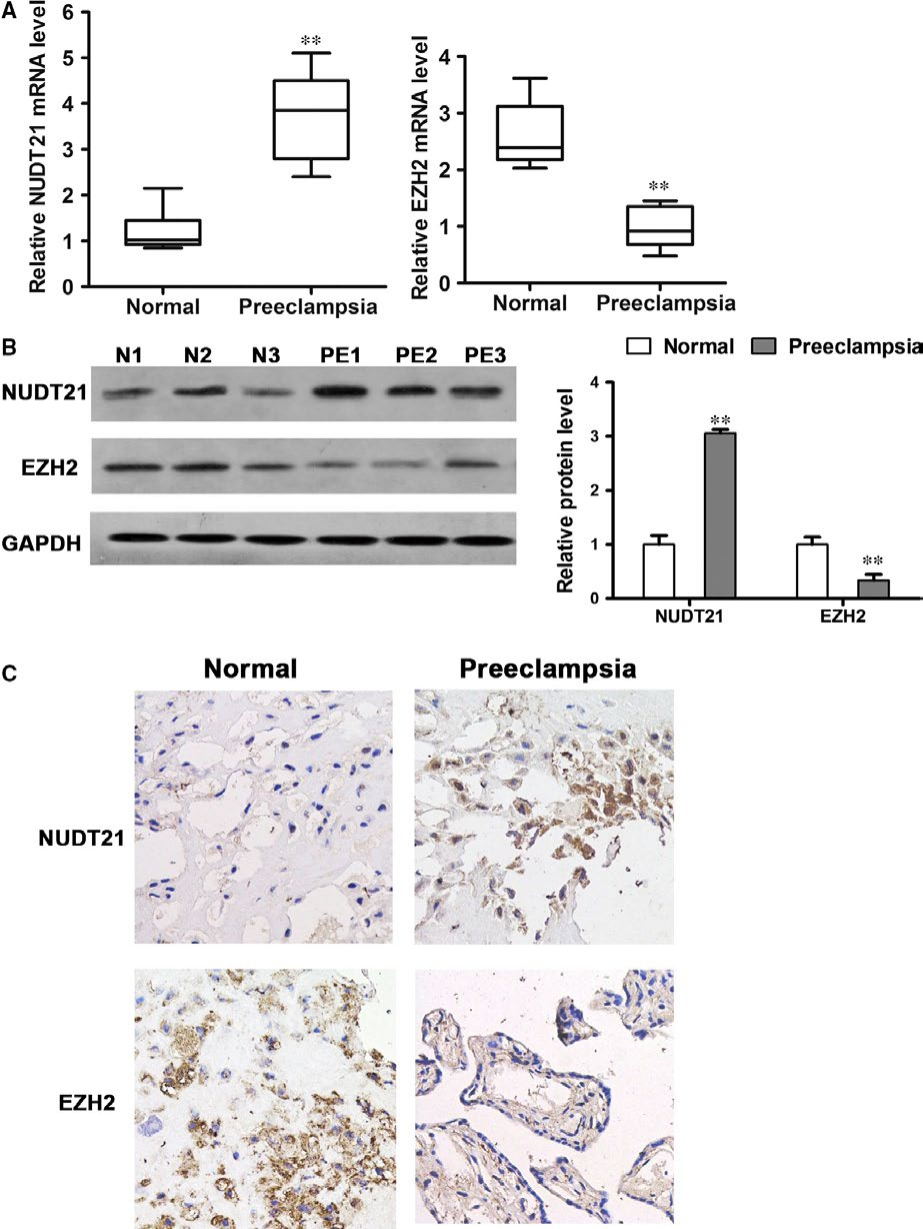
NUDT21 expression levels are increased in PE placental tissue. The relative expression levels of Nudt21 and EZH2 in paired placental tissues from PE women and women with normal pregnancies, as determined by qRT‐PCR (A), western blotting (B) and IHC (C) with anti‐NUDT21 and anti‐EZH2 antibodies. The data from at least three biological replicates are shown. Values are the mean ± SEM; **P < 0.01

The expression levels of NUDT21 and EZH2 were investigated in paired placental tissues from PE women and women with normal pregnancies by qRT‐PCR (Figure 1A). The mRNA levels of NUDT21 were increased (*P* < 0.01) and the levels of EZH2 were decreased (*P* < 0.01) in PE placental tissue (n = 20) compared with normal placental tissue (n = 20). Western blot analysis confirmed this pattern at the protein level, with NUDT21 being increased (*P* < 0.01) and EZH2 being decreased (*P* < 0.01) in PE placental tissue compared with paired normal tissue samples (Figure 1B). Furthermore, IHC of paired sections with anti‐NUDT21 and anti‐EZH2 primary antibodies revealed up‐regulated expression of NUDT21 and down‐regulated expression of EZH2 in PE placental tissue compared with control tissue (Figure 1C).

### 3.2 NUDT21 inhibits the proliferation and migration of trophoblast cells

**Figure 2:**
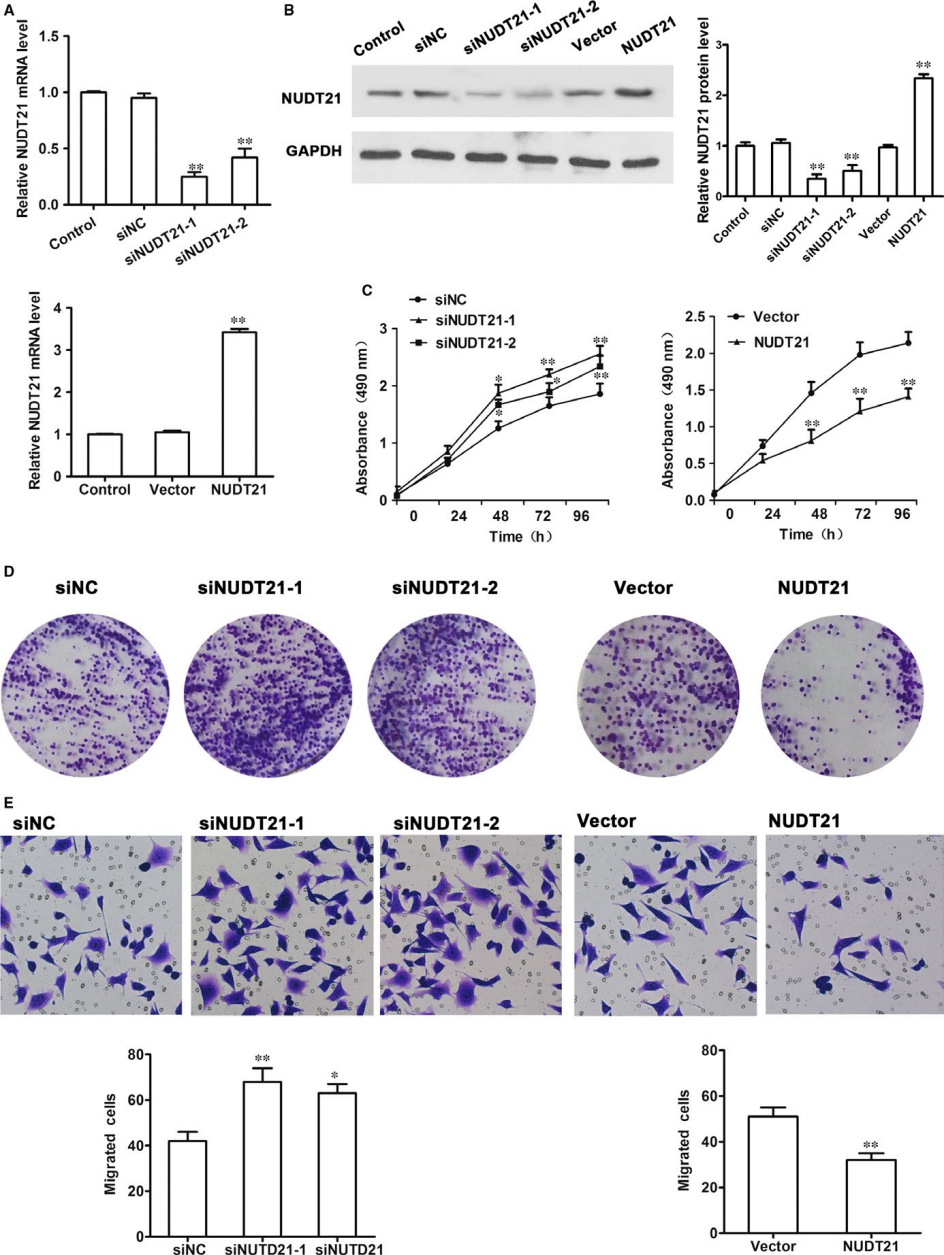
NUDT21 inhibits the proliferation and migration of trophoblast cells. To analyse the effects of NUDT21 expression, NUDT21 siRNA was transfected into the human trophoblast cell line, HTR‐8/SVneo, to generate the knocked down cell lines, NUDT21‐1 and NUDT21‐2, and pcDNA‐NUDT21 was transfected into HTR‐8/SVneo cells to generate the NUDT21 overexpressing cell line. The relative expression of Nudt21 was then measured by qRT‐PCR (A) and Western blotting (B). C, MTT assay to analyse cell proliferation over 96 h in the NUDT21 knockdown and overexpressing cell lines. D, Colony formation assays were implemented to assess the cell viability for NUDT21 knockdown and overexpressing in trophoblast cells. E, Migration assay to analyse the migratory ability of the NUDT21 knockdown and overexpressing cells. The data from at least three biological replicates are shown. Values are the mean ± SEM; **P < 0.01, *P < 0.05 compared with siNC‐transfected or vector only‐transfected cells

To analyse the effects of NUDT21 expression, NUDT21 siRNA was transfected into the human trophoblast cell line, HTR‐8/SVneo, and two knocked down cell lines, NUDT21‐1 and NUDT21‐2, were generated. NUDT21 expression was attenuated in both of the NUDT21‐1 and NUDT21‐2 cell lines compared with control cells at the mRNA (*P* < 0.01) and protein levels (*P* < 0.01), as confirmed by RT‐PCR and western blotting (Figure 2A,B). NUDT21 was also overexpressed in HTR‐8/SVneo cells by transfection with pcDNA‐NUDT21 (*P* < 0.01, compared with vector only cells), as confirmed by RT‐PCR and Western blot analysis (Figure 2A,B).

MTT assay was then performed to assess cell proliferation and the results revealed that knockdown of NUDT21 increased the growth rate of trophoblast cells, whereas overexpression of NUDT21 decreased the growth rate of these cells over a period (Figure 2C, D; compared with siNC or vector only cells). Similarly, a migration assay revealed that knockdown of NUDT21 increased the migratory activity of trophoblast cells (*P* < 0.01 for NUDT21‐1 and *P* < 0.05 for NUDT21‐2 compared with siNC‐transfected cells), whereas overexpression of NUDT21 decreased the migration of these cells (Figure 2E; *P* < 0.01 compared with vector only cells).

### 3.3 NUDT21 suppresses endothelial function in vitro

**Figure 3:**
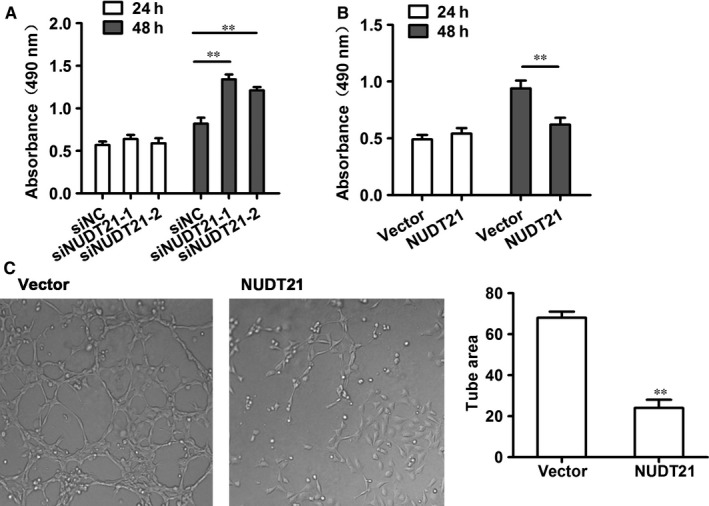
NUDT21 suppresses endothelial function in vitro. To confirm the effects observed in trophoblast cells in HUVEC cells, HUVEC cells were transfected with the siRNAs NUDT21‐1 and NUDT21‐2 for knockdown of NUDT21 and pcDNA‐NUDT21 for NUDT21 overexpression. A, MTT assay to analyse cell proliferation at 24 and 48 h in the NUDT21 knockdown and (B) overexpressing HUVEC cells. C, Tube formation assay in NUDT21 overexpressing HUVEC cells after 12 h. The figure shows representative pictures after transfection. Right panel presents the data from three independent experiments. Data are presented as the mean ± SEM. **P < 0.01 compared with siNC‐transfected (panel A) or vector only‐transfected (panels B, C) cells

Next, HUVEC cells were transfected with the siRNAs NUDT21‐1 and NUDT21‐2 for knockdown of NUDT21 and pcDNA‐NUDT21 for NUDT21 overexpression to confirm that the same effects were seen in HUVEC cells as HTR‐8/SVneo cells. Transfected HUVEC cells were subjected to an MTT assay to assess cell proliferation. No effects were seen at 24 hours; however, at 48 hours, knockdown of NUDT21 increased the growth rate of HUVEC cells (*P* < 0.01 compared with siNC‐transfected cells) (Figure 3A), whereas overexpression of NUDT21 decreased the growth rate of these cells (*P* < 0.01 compared with vector only cells) (Figure 3B), confirming the results in trophoblast cells. Next, the effect of NUDT21 on tube formation was assessed by transfecting HUVEC cells seeded in a 96‐well plate with NUDT21 then assaying tube formation after 12 hours (Figure 3C). The tube formation ability of NUDT21‐overexpressing HUVEC cells was significantly reduced compared with the vector only control cells (Figure 3D). (*P* < 0.01).

### 3.4 NUDT21 mediates EZH2 silencing by regulating the length of the 3'‐UTR of the *Ezh2* gene

**Figure 4:**
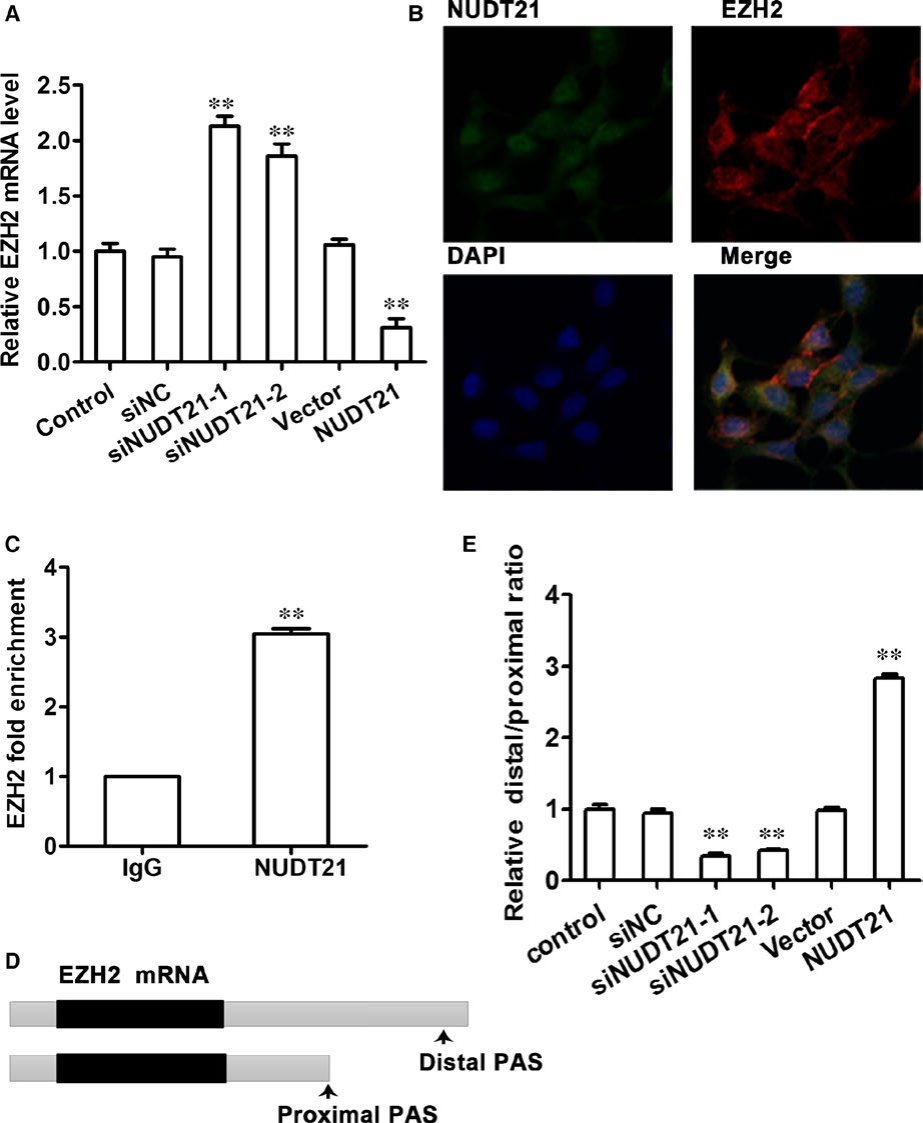
NUDT21 mediates EZH2 silencing by regulating the length of the 3'‐UTR of the *EZH2* gene. To investigate the regulatory effect of NUDT21 on EZH2, siNUDT21‐transfected and NUDT21‐overexpressing trophoblast cells were employed. A, qRT‐PCR analysis of siNUDT21‐transfected or NUDT21‐overexpressing trophoblast cells to analyse the mRNA levels of EZH2. B, IF staining was performed using appropriate anti‐NUDT21 and anti‐EZH2 antibodies to assess the distribution of NUDT21 (green) and EZH2 (red) in cells. C, RIP assay using NUDT21 antibody to confirm that EZH2 interacts with NUDT21. D, Schematic diagram of the 3’‐UTR sequences of the model gene. E, qRT‐PCR monitoring of the relative EZH2 sites used in siNUDT21‐transfected or NUDT21‐overexpressing cells. Data are presented as the mean ± SEM. **P < 0.01 compared with siNC‐transfected or vector only‐transfected cells

Here, we showed that NUDT21 is an interaction partner of EZH2. To investigate the regulatory effect of NUDT21 on EZH2, qRT‐PCR analysis of siNUDT21‐treated or NUDT21‐overexpressed trophoblast cells was performed and the mRNA levels of EZH2 were found to be altered (Figure 4A). Following knockdown of NUDT21, EZH2 expression was increased (*P* < 0.01 compared with siNC‐transfected cells) and conversely, overexpression of NUDT21 resulted in decreased expression of EZH2 (*P* < 0.01 compared with vector only transfected cells). IF staining was performed using appropriate anti‐NUDT21 and anti‐EZH2 antibodies to assess the distribution of NUDT21 (green) and EZH2 (red) in cells (Figure 4B). The merged image indicated co‐localization of these two proteins. An RIP assay using NUDT21 antibody then confirmed that EZH2 interacts with NUDT21 (Figure 4C). Finally, qRT‐PCR was performed to monitor the relative EZH2 sites used in siNUDT21‐treated or NUDT21‐overexpressed cells. Schematic diagram shows the 3’‐UTR sequences of the model gene (Figure 4D). Silencing of NUDT21 resulted in decreased use of the EZH2 distal site (*P* < 0.01 compared with siNC‐transfected cells), whereas overexpression of NUDT21 resulted in increased use of the distal site (*P* < 0.01 compared with vector only transfected cells) (Figure 4E).

### 3.5 NUDT21 increases the efficiency of miR‐mediated EZH2 silencing

**Figure 5:**
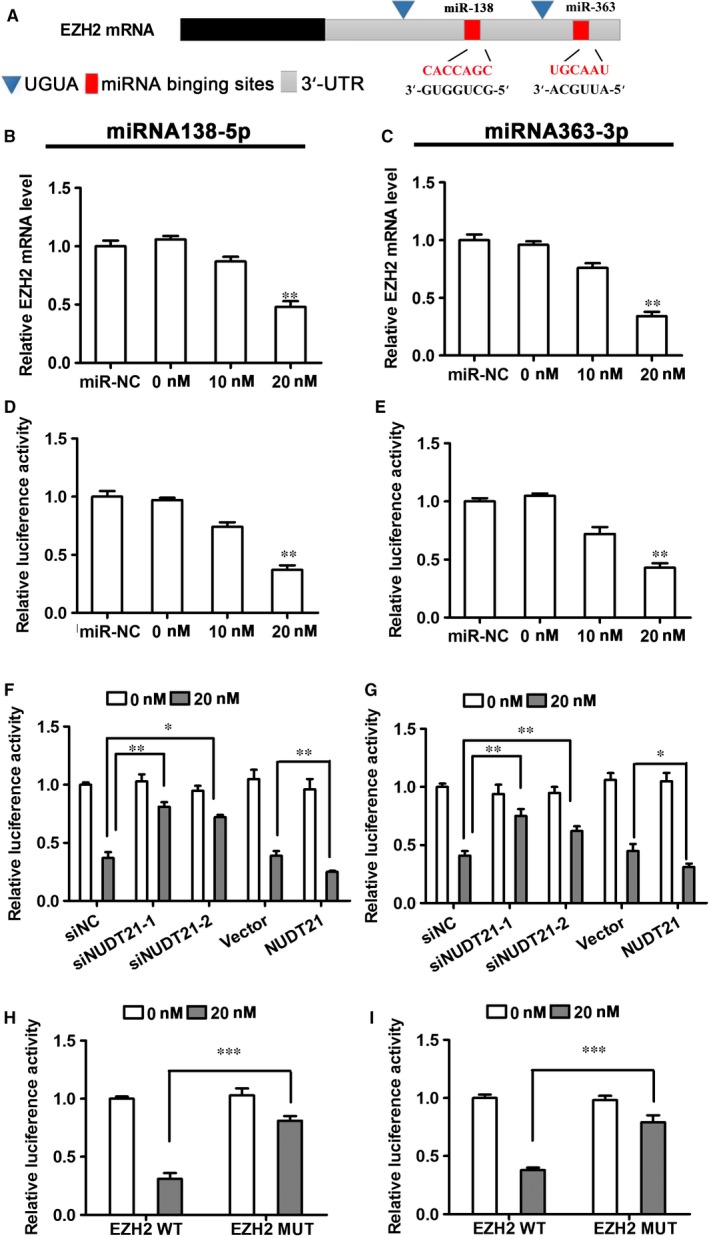
NUDT21 increases the efficiency of miRNA‐mediated gene silencing. HTR8/SVneo cells were transfected with miR‐138‐5p or miR‐363‐5p mimics (0, 10, 20 μg). A, Schematic diagram of UGUA sequence sites and miRNA binding sites in 3ʹ‐UTR of EZH2 mRNA. (B, C) qRT‐PCR analysis to determine the effects of miRNA binding on the mRNA expression of EZH2. (D, E) Luciferase reporter assay to confirm the effects of miRNA binding on EZH2 mRNA expression in the cells transfected with miRNA mimics. (F, G) Luciferase assays in NUDT21 knockdown and NUDT21 overexpressing cells to assess the effect on miRNA inhibition rates in both the miR‐138‐5p‐regulated and miR‐363‐5p‐regulated EZH2 luciferase reporter system. (H, I) An *Ezh2* mutant was generated in which the two TGTA sites recognized by NUDT21 were mutated to CAGT and a luciferase assay was performed to analyse the miRNA‐mediated effects on luciferase activity. Data are presented as the mean ± SEM. ***P < 0.001; **P < 0.01; *P < 0.05 compared with the relevant controls

By sequence analysis, we detected miRNA binding sites, including miR138 and miR363, within the EZH2 gene. Schematic diagram shows the UGUA sequence sites and miRNA binding sites in 3’‐UTR of EZH2 mRNA (Figure 5A). HTR8/SVneo cells were transfected with miR‐138‐5p or miR‐363‐5p mimics and qRT‐PCR analysis was performed to determine the effects of miRNA binding on EZH2 expression. At a 20 µg concentration of miRNA mimic, miR‐138‐5p (Figure 5B) and miR‐363‐5p (Figure 5C), EZH2 mRNA levels were significantly decreased (*P* < 0.01 compared with miR‐NC), indicating the miRNA‐mediated gene silencing of *Ezh2*. These findings were confirmed by the relative luciferase activities in cells transfected with the miRNA mimics (Figure 5D,E). Further luciferase assays in NUDT21 knockdown cells demonstrated that miRNA inhibition rates were repressed, whereas miRNA inhibition rates were enhanced after NUDT21 overexpression in both the miR‐138‐5p‐regulated and miR‐363‐5p‐regulated EZH2 luciferase reporter systems (Figure 5F,G, respectively). Finally, a *Ezh2* mutant was generated in which the two TGTA sites recognized by NUDT21 were mutated to CAGT, as previously reported.^13^ A luciferase activity assay then revealed that the miRNA‐mediated inhibition of luciferase activity was abolished after the UGUA sequences in the 3'‐UTR had been mutated (*P* < 0.1 compared with *Ezh2* wild‐type) (Figure 5H,I). In summary, NUDT21 enhanced the efficiency of miRNA‐mediated gene silencing by extending the 3'‐UTR of EZH2 (by exposing more miRNA binding sites, including miR138 and miR363), thereby increasing the efficiency of EZH2 binding.

### 3.6 EZH2 is important for trophoblast cells and endothelial cell function

**Figure 6:**
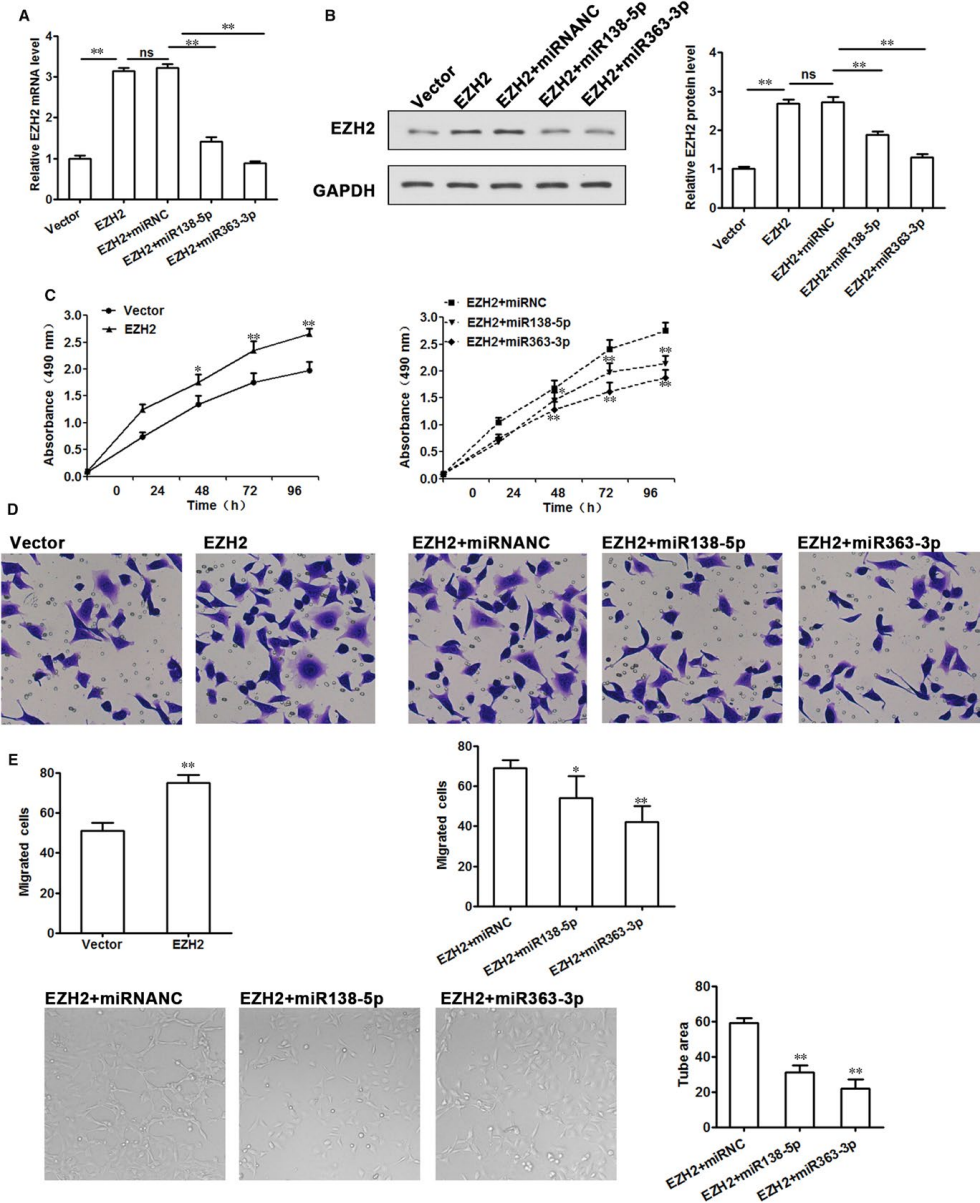
EZH2 is important for trophoblast growth and migration and endothelial cell tube formation. pcDNA‐EZH2 was transfected into HTR‐8/SVneo cells to generate the EZH2 overexpressing cell line. After 24 h, EZH2 overexpressing HTR8/SVneo cells were transfected with miR‐138‐5p or miR‐363‐3p mimics to down‐regulated EZH2 expressing. A, qRT‐PCR analysis and (B) WB analysis of EZH2‐overexpressing or miR‐mediated EZH2 overexpressing trophoblast cells to analyse the mRNA levels of EZH2. C, MTT assay to analyse cell proliferation over 96 h in the EZH2 overexpressing cell lines and miRNAs can inhibit cell proliferation caused by EZH2 overexpression. D, Migration assay to analyse the migratory ability of the EZH2 overexpressing cells and miRNAs can inhibit migration caused by EZH2 overexpression. E, HUVEC cells were transfected with pcDNA3.1‐EZH2+ miRNANC, pcDNA3.1‐EZH2+miR‐138‐5p mimic, pcDNA3.1‐EZH2+miR‐138‐5p mimic. The left pictures show representative result. The right bar chart is the statistic analysis. The results reveal that miRNAs can inhibit HUVEC angiogenesis caused by EZH2 overexpression. The data from at least three biological replicates are shown. Values are the mean ± SEM; **P < 0.01; *P < 0.05 compared with the relevant controls

To investigate the biological effects of EZH2 on development PE, we analysed the growth rates and migration of both the Vector and EZH2 overexpression cell lines and tube formation ability of HUVEC cells. EZH2 was also overexpressed in HTR‐8/SVneo cells by transfection with pcDNA‐EZH2 (*P* < 0.01, compared with vector only cells), as confirmed by RT‐PCR and Western blot analysis. MiRNAs bind to target gene mRNAs for either mRNA degradation or translation repression. To investigate how miRNAs modulates EZH2 expression, we also tested effects of miR‐138‐5p and miR‐363‐5p on EZH2 mRNA/protein levels in HTR‐8/SVneo cells. The result show that EZH2 mRNA/protein levels were up‐regulated by EZH2 overexpression and down‐regulated by miR‐138‐5p and miR‐363‐5p (Figure 6A,B).

MTT assays showed that the growth rates of the EZH2 overexpression cell lines were increased compared with those of the vector control, while the growth rates of the miR‐138‐5p and miR‐363‐5p mediated EZH2 overexpression cell lines were decreased compared with those of the EZH2 overexpression in HTR‐8/SVneo cells (Figure 6C). We also analysed the migration of these cell lines to determine the role of EZH2 in cell migration. The number of cells migrating through transwell pores was significantly promoted after EZH2 overexpression. As expected, miR‐138‐5p and miR‐363‐3p significantly reduced EZH2 overexpression HTR‐8/SVneo cell migration compared to the corresponding miRNANC(negative control) cells(Figure 6D).

Next, HUVEC cells were transfected with the pcDNA‐EZH2 for EZH2 overexpression and after 24 hours the cells transfected with miR‐138‐5p or miR‐363‐3p mimics for EZH2 repression. At 48 hours, miR‐138‐5p or miR‐363‐3p‐mediated repression of EZH2 decreased the tube formation ability compared with the EZH2 overexpression cells(Figure 6E).These results suggested that miR‐138‐5p or miR‐363‐3p suppresses trophoblast growth and migration and tube formation ability at least partially by directly inhibiting EZH2. EZH2 affects placental development and its dysregulation contributes to PE development.

## 4 DISCUSSION

It has previously been established that NUDT21 plays a role in APA and is a key regulator of 3'‐UTR length.^13^ Down‐regulation of *Nudt21* expression in cells has been reported to lead to changes in alternative poly(A) site utilization for several somatic mRNAs.^16^, ^17^ Many studies have established that Ezh2 acts as a suppressor of RNA transcription through H3K27me3, include in PE.^28^ In this study, we investigated the mechanistic basis for the marked increase in NUDT21 expression in the placentas of pregnant women with PE compared with normal pregnancies. Our research confirmed the interaction between EZH2 and NUDT21 and showed that this interaction plays an important role in the crosstalk between APA and miRNA‐mediated gene silencing in PE. Knockdown of NUDT21 in HTR‐8/SVneo cells suppressed the proliferative and migratory activity of these trophoblasts and also inhibited tube formation. Based on our findings, we propose a simplified model of the relationship between NUDT21 and miRNA‐mediated gene silencing in PE whereby NUDT21 elongates the 3'‐UTR of mRNAs by APA thereby exposing more miRNA binding sites and enhancing the efficiency of miRNA‐mediated gene silencing by promoted EZH2 binding, contributing to PE development and progression.

This model for the relationship between NUDT21 and miRNA‐mediated gene silencing is similar to that described in previous studies,^11^ particularly a recent parallel study by Sun and colleagues in HCC.^13^ They confirmed the role of NUDT21 in APA and miRNA‐mediated gene silencing. Similar to our findings, NUDT21 was shown to elongate the 3'‐UTR of mRNA and enhance the efficiency of miRNA‐mediated gene silencing by increasing the efficiency of AGO2‐mRNA binding, which promoted cell proliferation. In this case, loss of NUDT21 shortened the 3'‐UTR of various oncogenes in HCC cells, effectively removing miRNA binding sites and enabling these genes to evade miRNA regulation, become overexpressed and induce tumorigenesis and metastasis.

Various mechanisms, including abnormal transcriptional control of miRNAs, amplification or deletion of miRNA genes, defects in the miRNA biogenesis machinery and dysregulated epigenetic changes, can explain the abnormal expression in PE. For example, miR‐15 family members, such as miR‐424 and miR‐16, play a role in placentation. miR‐363 plays an important role in post‐transcriptional regulation of CM differentiation.^29^, ^30^ A recent study reported that luciferase reporter assay identified enhancer of zeste homologue 2 (EZH2) as a direct target gene of miR‐138, and the protein expression of EZH2 was negatively regulated by miR‐138 in Hep‐2 cells.^31^ We demonstrated that miR‐138‐5p and miR‐363‐3p suppresses cytotrophoblast growth and migration at least partially by directly targeting EZH2.

However, the mechanisms responsible for the differences in the functions of these miRNAs in placentation are largely unknown. As previous studies have demonstrated that APA was correlated with the efficiency of miRNA‐mediated gene silencing.^11^ In this study, we found that NUDT21 interacts with EZH2. And therefore, we speculated that the interaction between NUDT21 and EZH2 might play important role in the cross talk between APA and miRNA‐mediated gene silencing in PE.

Mediative effect of NUDT21 on different bioactivity in process of post‐transcription has been well established. Knockdown of NUDT21, a master 3’UTR‐shortening regulator, represses tumour‐suppressor genes such as PHF6 and LARP1 in trans in a miRNA‐dependent manner.^32^ NUDT21 knockdown increases usage of the proximal PAP site in the PSMB2 and CXXC5 3’UTRs, resulting in marked increase in the expression of PSMB2 and CXXC5.^33^ NUDT21 regulates MeCP2 protein quantity in neuropsychiatric patient‐derived lymphoblastoid cells and elevated NUDT21 increases usage of the distal polyadenylation site in the MECP2 3ʹ UTR, resulting in an enrichment of inefficiently translated long mRNA isoforms.^34^ In this study, we have demonstrated that NUDT21 and EZH2 we co‐localized in trophoblast cell, and silencing of NUDT21 resulted in decreased use of the EZH2 distal site, whereas overexpression of NUDT21 resulted in increased use of the distal site.

There is debate as to how representative the HTR8/SVneo cell line is of trophoblast cells in vivo. Morales‐Prieto et al revealed that HTR8/SVneo cells closely resemble primary trophoblast cells in many aspects but discrepancies were identified in terms of their miRNA profiles.^35^ Further work is needed to validate the results obtained in HTR8/SVneo cells in primary cells.

Our findings indicate NUDT21 is up‐regulated in placental samples from PE patients compared with levels in controls. Our results demonstrate for the first time that NUDT21 plays an important role in PE. Functional studies showed that NUDT21 elongated the 3'‐UTR of mRNAs thereby exposing more miRNA binding sites (including miR138 and miR363), which enhanced the efficiency of miRNA‐mediated gene silencing and promoted EZH2 binding. Based on these collective results presented above, we propose that NUDT21 bring into play functions and may be a valuable strategy for pre‐eclamptic for further basic study and cell‐therapy applications.

## CONFLICT OF INTEREST

The authors declare no conflict of interest.
